# Increased expression of native cytosolic Cu/Zn superoxide dismutase and ascorbate peroxidase improves tolerance to oxidative and chilling stresses in cassava (*Manihot esculenta* Crantz)

**DOI:** 10.1186/s12870-014-0208-4

**Published:** 2014-08-05

**Authors:** Jia Xu, Jun Yang, Xiaoguang Duan, Yueming Jiang, Peng Zhang

**Affiliations:** 1 Shanghai Chenshan Plant Science Research Center, Chinese Academy of Sciences, Shanghai Chenshan Botanical Garden, 3888 Chenhua Road, Shanghai 201602, China; 2 National Key Laboratory of Plant Molecular Genetics and National Center for Plant Gene Research (Shanghai), Institute of Plant Physiology and Ecology, Shanghai Institutes for Biological Sciences, Chinese Academy of Sciences, Shanghai 200032, China; 3 Key Laboratory of Plant Resources Conservation and Sustainable Utilization, South China Botanical Garden, Chinese Academy of Sciences, Guangzhou 510650, China

**Keywords:** Manihot esculenta Crantz, Cytosolic superoxide dismutase, Cytosolic ascorbate peroxidase, Reactive oxygen species scavenging, Abiotic stress resistance

## Abstract

**Background:**

Cassava (*Manihot esculenta* Crantz) is a tropical root crop, and is therefore, extremely sensitive to low temperature; its antioxidative response is pivotal for its survival under stress. Timely turnover of reactive oxygen species (ROS) in plant cells generated by chilling-induced oxidative damages, and scavenging can be achieved by non-enzymatic and enzymatic reactions in order to maintain ROS homeostasis.

**Results:**

Transgenic cassava plants that co-express cytosolic superoxide dismutase (SOD), *Me*Cu/ZnSOD, and ascorbate peroxidase (APX), *Me*APX2, were produced and tested for tolerance against oxidative and chilling stresses. The up-regulation of *Me*Cu/ZnSOD and *Me*APX2 expression was confirmed by the quantitative reverse transcriptase-polymerase chain reaction, and enzymatic activity analyses in the leaves of transgenic cassava plant lines with a single-transgene integration site. Upon exposure to ROS-generating agents, 100 μM ROS-generating reagent methyl viologen and 0.5 M H_2_O_2_, higher levels of enzymatic activities of SOD and APX were detected in transgenic plants than the wild type. Consequently, the oxidative stress parameters, such as lipid peroxidation, chlorophyll degradation and H_2_O_2_ synthesis, were lower in the transgenic lines than the wild type. Tolerance to chilling stress at 4°C for 2 d was greater in transgenic cassava, as observed by the higher levels of SOD, catalase, and ascorbate-glutathione cycle enzymes (e.g., APX, monodehydroascorbate reductase, dehydroascorbate reducatase and glutathione reductase) and lower levels of malondialdehyde content.

**Conclusions:**

These results suggest that the expression of native cytosolic SOD and APX simultaneously activated the antioxidative defense mechanisms via cyclic ROS scavenging, thereby improving its tolerance to cold stress.

## Background

Cassava (*Manihot esculenta* Crantz) is an important tropical root crop that plays an important role not only in ensuring food security, but also in various bioindustrial applications such as animal feed, modified starch and biofuels [1],[2]. Because cassava is native to tropical regions, it is extremely cold-sensitive; its growth is drastically affected at temperatures lower than 18°C, and the plant cannot survive for long after exposure to freezing conditions [3]–[5]. Therefore, low temperatures and freezing conditions are the most important limiting factors for the cultivating location, as well as productivity. The damage of apical shoot seems be to more critical than other parts of the cassava plant [5]. Improving the tolerability of the cassava plant to multiple stresses has therefore, become a major objective of cassava breeders, especially in subtropical regions [4],[5]. Under cold conditions, up-regulation of reactive oxygen species (ROS) turnover and scavenging in cassava has been reported, and therefore, genetic manipulation of intracellular ROS level might be an effective approach in improving tolerance to abiotic stresses in this tropical crop [5]–[7].

In the ROS scavenging system that is responsible for homeostasis in plant cells, superoxide dismutases (SODs, EC 1.15.1.1), enzymes that catalyze the dismutation of superoxide into oxygen and hydrogen peroxide, provide the first line of defense against ROS in various subcellular compartments, i.e. chloroplast, mitochondria and cytosol [8]. Essentially, there are three types of SODs, each containing either manganese, iron, or copper plus zinc as a prosthetic group [9]. Along with other ROS scavenging mechanisms like catalase (CAT; EC 1.11.1.6), glutathione peroxidases (GPXs) and peroxiredoxin reductases (PrxRs), and the ascorbate–glutathione (ASC-GSH) cycle, the ROS levels are maintained in a homeostatic state. In the ASC-GSH cycle, using ascorbate as an electron donor, ascorbate peroxidase (APX, EC 1.11.1.1) scavenges potentially harmful hydrogen peroxide to water from the chloroplasts and mitochondria, as well as other organelles [10],[11]. Therefore, the formation of toxic hydroxyl radicals by superoxide and hydrogen peroxide can be controlled by the combined enzymatic actions of SOD and APX [12].

Transgenic plants that express SOD or APX have shown enhanced tolerance to multiple stresses [13]. For example, over-expression of different SODs (FeSOD, MnSOD or Cu/ZnSOD) in transgenic plants of tomato, rice, poplar, alfalfa, etc., showed increased tolerance to methyl viologen (MV), ozone, high salinity, chilling or other stresses [14]–[17]. Transgenic plants have also demonstrated an increased tolerance against various abiotic stresses by the expression of either cytosolic- or organelle-targeted cytosolic APX [18]–[22]. However, some reports suggest no change in response to oxidative or environmental stress with the expression of a single antioxidant enzyme [23],[24]. These contradictory findings may be due to the complex network of plant antioxidant defenses, which possibly confer a higher tolerance to oxidative stress by pyramiding or stacking of multiple genes in a single genotype [25]. The gene-stacking approach entails manipulation of two or more desirable enzymes mediating the ROS turnover and scavenging pathways, in improving the abiotic stress tolerance in plants.

Indeed, co-expression of two distinct ROS-scavenging enzymes, such as SOD and other ROS-scavenging enzymes, in the chloroplasts or cytosol in transgenic plants has a synergistic effect in increasing the levels of abiotic stress resistance. For example, coupled expression of Cu/ZnSOD and APX in transgenic plants of *Festuca arundinacea*, potato, tobacco, sweet potato and plum led to increased tolerance to multiple abiotic stresses, e.g., the herbicide methyl viologen (MV), chilling, high temperature and drought [12],[26]–[29]. Payton et al. [30] showed that co-expression of glutathione reductase (GR, EC 1.6.4.2) and APX in cotton improved antioxidant enzyme activity during moderate chilling at high light intensity, in chloroplasts [30]. Co-expression of the *Suaeda salsa* glutathione S-transferase (GST) and CAT1 in rice also caused tolerance to stresses caused by salt and paraquat [31]. Taken together, these data indicated that the combination of transgenes encoding different ROS-scavenging enzymes in various subcellular compartments might have a synergistic effect in improving stress tolerance.

Lately, plant breeders and biotechnologists have appreciated the molecular insights and advances in cassava abiotic stress resistance, on a global scale. Apart from the various approaches from traditional breeding to field evaluation [32], studies of cassava response to drought or cold stress at the molecular level have reportedly used the “omics” technology, e.g., expressed sequence tags, cDNAs and oligonucleotide microarray [5],[33]–[38]. However, few studies on improved tolerance to environmental stresses using genetic engineering have been reported [7],[39]. Senescence-induced expression of the isopentenyl transferase gene in cassava showed increased drought resistance, as observed by the elevated content of cytokinin in mature leaves, and prolonged leaf life [39]. Enhanced ROS scavenging by simultaneous expression of cytosolic *Me*Cu/ZnSOD and peroxisomal *Me*CAT1 in transgenic cassava also confirmed the improved tolerance towards drought and cold temperatures [7].

In the present study, transgenic cassava plants co-expressing both cytosolic *Me*Cu/ZnSOD (Genbank accession No. AY642137) and cytosolic *Me*APX2 (GenBank accession No. AY973622) showed enhanced ROS scavenging capacity, thereby leading to enhanced tolerance to oxidative stresses that was induced by MV, H_2_O_2_ as well as chilling. Our results suggest that manipulation of ROS-scavenging enzyme systems by the overexpression of both cytosolic SOD and APX is a worthwhile approach to produce transgenic plants with enhanced tolerance to a wide range of abiotic stresses.

## Results

### Subcellular localization of *Me*Cu/ZnSOD and *Me*APX2

Green fluorescent protein (GFP) was the fusion protein used for subcellular localization of *Me*Cu/ZnSOD and *Me*APX2. The control, CaMV 35S-eGFP construct, exhibited GFP fluorescence in the cytoplasm and nucleus of agroinfiltrated cells of *N. benthamiana* leaves. The *Me*Cu/ZnSOD has been reported as a cytosolic SOD isoform in our previous report [7]. The *Me*APX2 was located in the cytosol of the leaf cells, as indicated by the fused GFP fluorescence (Additional file 1) that was consistent with the signal peptide sequences predicted by Reilly et al. [38]. Therefore, both enzymes used in the study were cytosolic proteins.

### Molecular characterization of the transgenic plants

**Figure 1:**
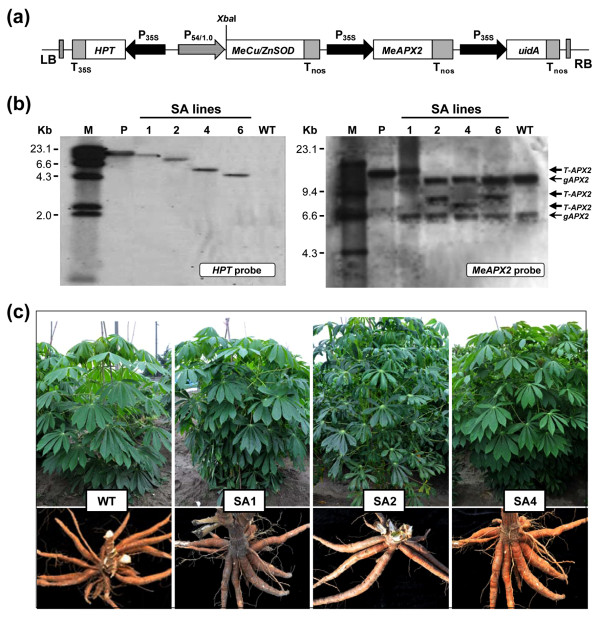
**Molecular and phenotypic analysis of SA transgenic cassava coupled expression of cytosolic*Me*Cu/ZnSOD and cytosolic*Me*APX2 genes. (a)** Schematic presentation of the T-DNA region of pC-P54::MeCu/ZnSOD-35S::MeAPX2 with unique *Xba*I site. LB, left border; RB, right border of T-DNA; T_35S_, CaMV 35S terminator; T_NOS_, NOS terminator ; P_35S_, cauliflower mosaic virus 35S promoter; P_54/1.0_, vascular-specific promoter p54/1.0; HPT, hygromycin phosphotransferase. **(b)** Southern blot analysis of transgenic and WT cassava plants for transgene integration. Transgene integration patterns in SA lines detected *Xba*I-digested genomic DNA by *HPT* (left panel) and *MeAPX2* (right panel) probes. gAPX, genomic DNA of cassava ascorbate peroxidase; T-APX2, transgene *Me*APX2; M, λ *Hin*dIII DIG-labeled molecular marker; P, plasmid pC-P54::MeCu/ZnSOD-35S::MeAPX2; WT, wild-type control. Numbers indicate different transgenic lines. **(c)** Plant growth status and phenotype evaluation in field. WT, wild-type control; SA1, SA2 and SA4, independent transgenic cassava plant lines.

Four independent transgenic plant lines (named SA1, SA2, SA4 and SA6) harboring the *MeCu/ZnSOD* and *MeAPX2* gene-expressing cassettes (Figure 1a) were produced by the use of *Agrobacterium*-mediated transformation and subcultured *in vitro* regularly. Confirmation of the single integration event of the pC-P54::MeCu/ZnSOD-35S::MeAPX2 T-DNA in these transgenic lines were carried out by the Southern blotting technique using *Xba*I-digested cassava genomic DNA, which were extracted from leaves of *in vitro* plants and hybridized with DIG-labeled *HPT* probe (Figure 1b, left panel). No signals were detected in the wild-type (WT) plants. However, when these WT plants were hybridized with the *MeAPX2* probe (Figure 1b, right panel), two bands of ~13 kb and ~6.6 kb were detected, indicating the possibility of two *APX* homologs in the cassava genome. The transgenic SA lines showed an additional band in their hybridization pattern, confirming that the transgenic plants were integrated at a single site (Figure 1b, right panel). All transgenic plants except SA2 line thrived successfully in the greenhouse and field, with a relatively normal leaf phenotype, growth capacity and root tuberization, similar to WT plants (Figure 1c); the SA2 line had a slightly dark-green and curled leaves when cultivated in the field. No significant differences of yield were found between WT and transgenic plant lines (Additional file 2).

**Figure 2:**
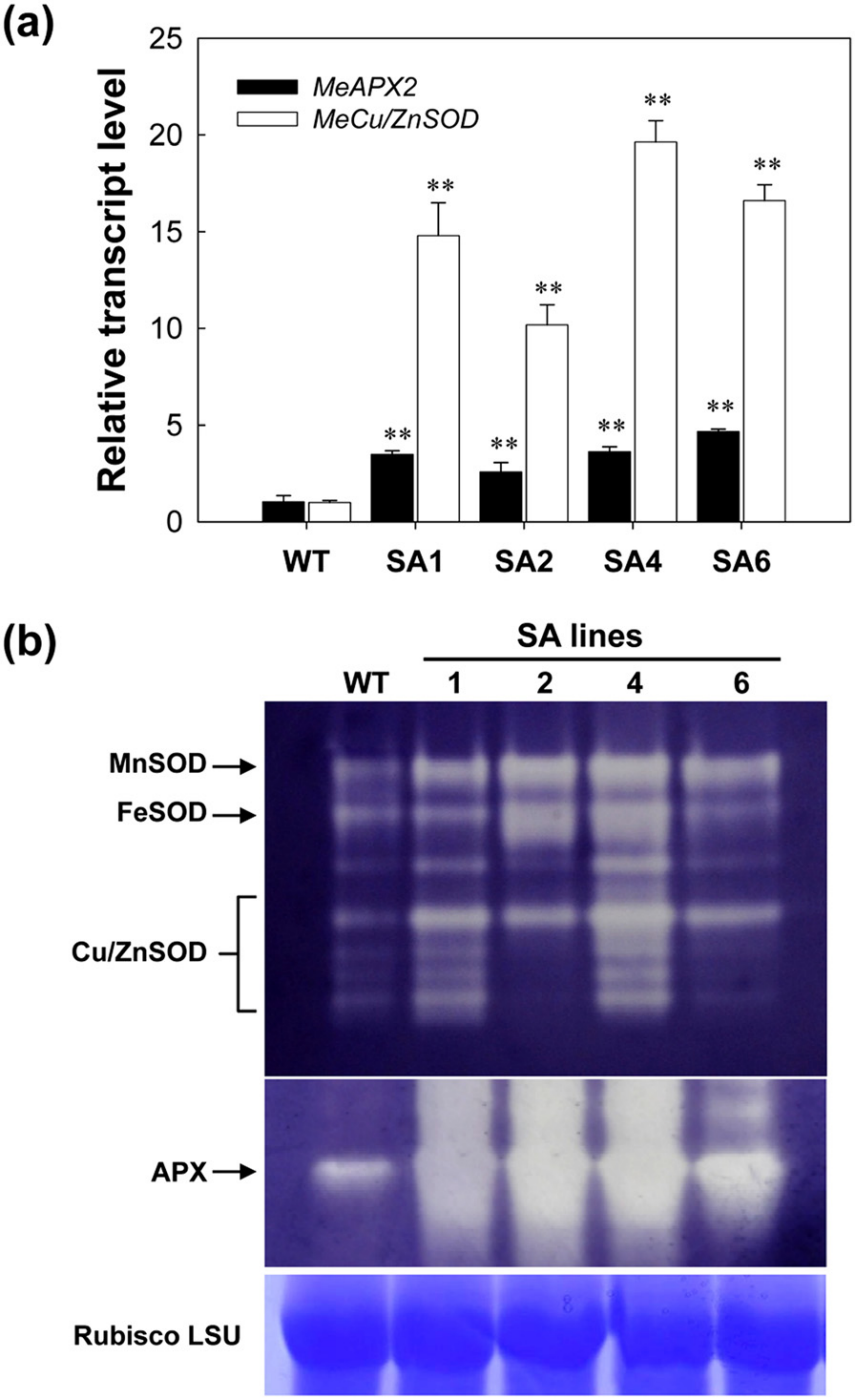
**Transcriptional and protein activity characterization of the SA transgenic plants. (a)** qRT-PCR analysis of *MeCu/ZnSOD* and *MeAPX2* expression levels both in WT and SA transgenic cassava lines. Total RNA was extracted from leaves of *in vitro* plants and the data are shown relative to the WT, using β-actin as an internal control. Data are presented as mean ± SD of three independent RNA samples. **(b)** SOD and APX isoforms in greenhouse-grown leaves of WT and transgenic plants detected by staining of non-denaturing polyacrylamide gel. Three SOD isoforms, MnSOD, FeSOD and Cu/ZnSOD, are indicated. The Rubisco LSU protein was used as a loading control. WT, wild-type control; SA with numbers, independent transgenic plant lines.

The basic transcriptional levels of *MeCu/ZnSOD* and *MeAPX2* in cassava leaves were confirmed by quantitative reverse transcriptase-polymerase chain reaction (qRT-PCR) (Figure 2a). The expressions of *MeCu/ZnSOD* and *MeAPX2* in transgenic lines were higher by up to 20- and 5-fold, respectively, when compared to that of WT plants. SOD expression level was about 5-times higher than APX2 in the SA lines, indicating that the *p54* promoter is stronger than CaMV 35S promoter in cassava, which is in agreement with the previous report [40]. Further, the changes in SOD and APX isoenzyme activity from leaves on non-denaturing gel were also confirmed (Figure 2b). A characteristic SOD isoenzyme banding pattern was found in all cassava leaves with Cu/ZnSOD, MnSOD and FeSOD [6], but the intensity of SOD bands was higher in all transgenic lines than the WTs. Similarly, a stronger APX isoenzyme band was also observed in all transgenic lines than that of WT (Figure 2b). These results indicate that the heightened production of SODs and APXs were achieved because of the transgene expression in transgenic cassava.

### Higher protoplast viability and mitochondrial integrity of mesophyll cells under H_2_O_2_ stress

**Figure 3:**
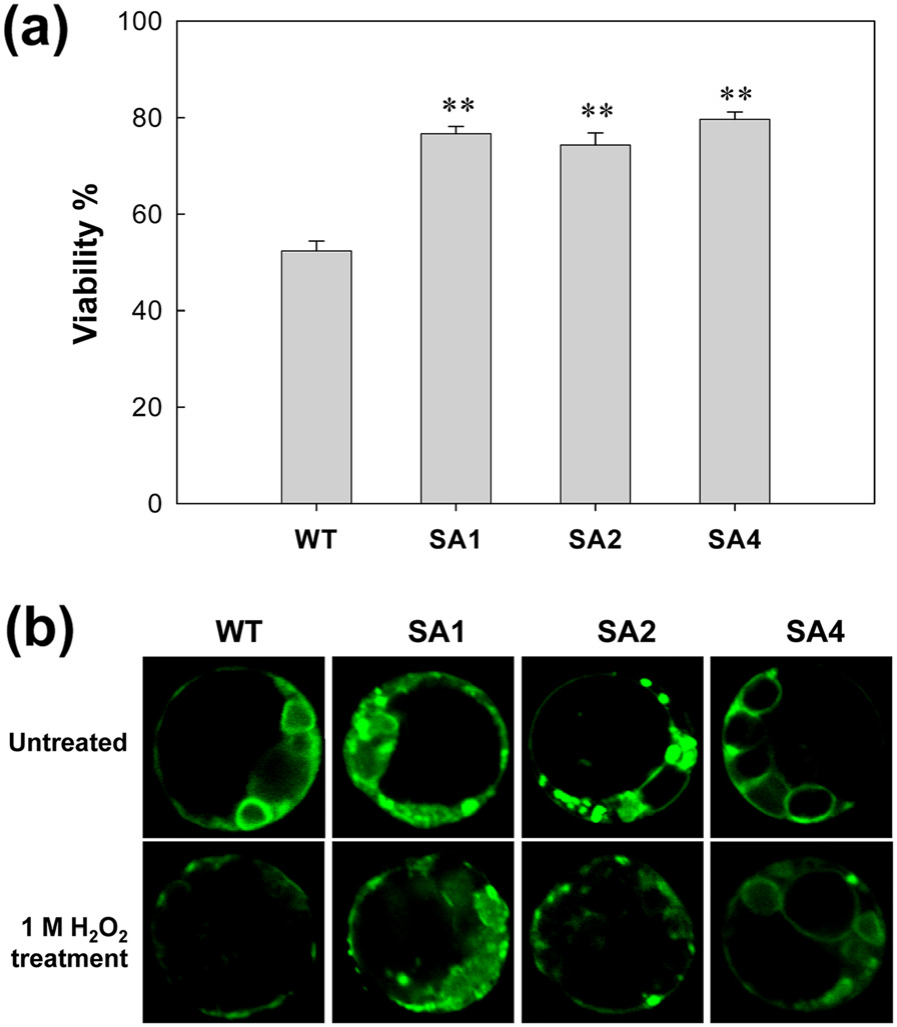
**Changes in protoplast viability and mitochondrial membrane integrity of cassava in the presence of 1 M H**_**2**_**O**_**2**_**. (a)** Viability of cassava mesophyll protoplasts after H_2_O_2_ treatment was estimated by fluorescein diacetate (FDA) staining. Data is represented as the mean value of six replicates ± SD (more than 300 cells were counted for each experiment per genotype). **Significant at 1% level from WT by *t-test*. **(b)** Mitochondrial membrane integrity under 1 M H_2_O_2_ stress. Cassava protoplasts were stained with the rhodamine 123 (Rh123) and the fluorescent signal was observed under a confocal microscope. Scale bar = 5 μm. WT, wild-type control; SA1, SA2 and SA4, independent transgenic plant lines.

Cell death and loss of mitochondrial integrity are indicators of stress damage. The viability of extracted transgenic and WT mesophyll protoplasts was above 95%, when stained by fluorescein diacetate (FDA). After 1 M H_2_O_2_ treatment, the viability of transgenic protoplasts from SA1, SA2, and SA4 decreased to 77%, 74%, and 80%, respectively, but the WTs showed 52% (Figure 3a), indicating significant improvement to stress tolerance. The mitochondrial integrity of mesophyll cells were observed by rhodamine 123 (Rh 123) staining. Strong Rh 123 fluorescent signals were observed in all cassava cells prior to H_2_O_2_ treatment, indicating normal mitochondrial activity in these cells. After H_2_O_2_ treatment, the protoplasts of the WT plants displayed diffuse and much weaker fluorescent signals, but the protoplasts derived from the transgenic plants retained their intense fluorescence activity (Figure 3b). The difference between transgenic lines and WT was evident, which indicated that the cells in transgenic plants displayed higher H_2_O_2_ tolerance than that in WT plants.

### Enhanced tolerance to H_2_O_2_-mediated oxidative stress in transgenic leaves

**Figure 4:**
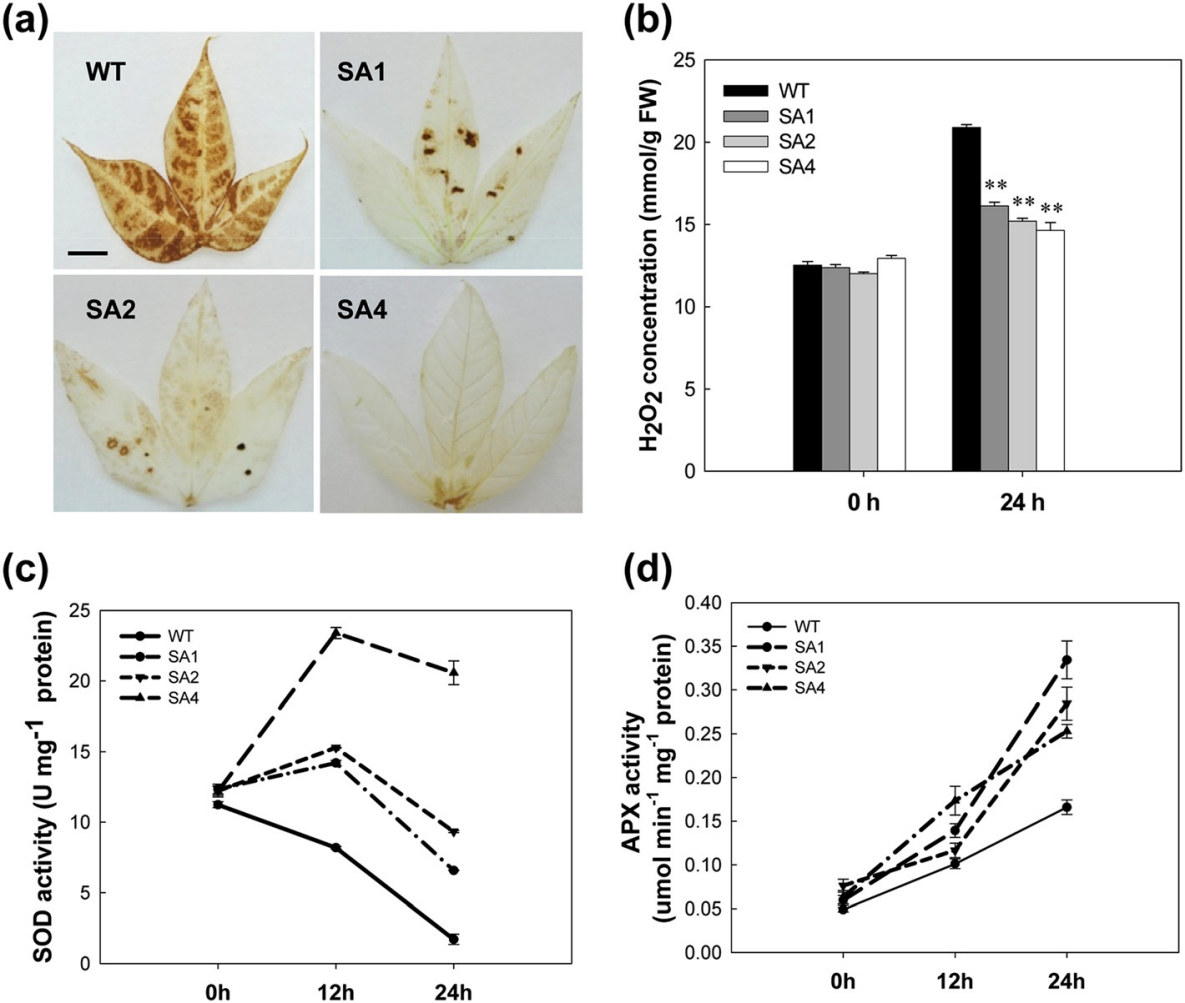
**Enhanced tolerance to H**_**2**_**O**_**2**_**-mediated oxidative stress in SA transgenic leaves. (a)** H_2_O_2_ accumulation in leaves detected by 3,3′-diaminobenzidine (DAB) staining. Scale bar = 0.5 cm. **(b)** Changes in levels of H_2_O_2_ concentration between WT and transgenic cassava during 0.5 M H_2_O_2_ treatment. **(c)** and **(d)** Changes in SOD and APX activities between WT and transgenic cassava during H_2_O_2_ treatment. WT, wild-type control; SA1, SA2 and SA4, independent transgenic plant lines. Values represent the means of three independent experiments ± SD. **Significant at 1% level from WT by *t-test*.

To evaluate the response of transgenic plants to oxidative stress, leaves showing a basal level of H_2_O_2_ production, as indicated by 3,3′-diaminobenzidine (DAB) staining (data not shown), were selected and treated with 0.5 M H_2_O_2_. After 24 hours, H_2_O_2_ molecules significantly accumulated in WT leaves (Figure 4a) at a concentration of 20.8 mmol/g, which was 1.6-times higher than that in the untreated leaves (Figure 4b). Leaves of transgenic plants showed much less H_2_O_2_ accumulation (Figure 4a), as observed by the elimination of the reaction product, DAB-H_2_O_2_, from a majority of leaves from the SA4 plants (Figure 4b). For example, the H_2_O_2_ concentration in the leaves of SA4 was 14.6 mmol/g, which was 11.6% higher than that in the untreated leaves.

The activities of SOD and APX in cassava leaves were assessed under normal and stressful conditions (Figure 4c, d). Enzyme activities were not significantly different between WT and transgenic plants under normal conditions. However, after stress induction, the SOD activity of WT plants decreased to 20% of the baseline value in 24 hours (1.7 U mg^−1^ protein, Figure 4c). In contrast, the SOD activity of all transgenic plant lines increased in 12 hours; for example, the activity level in SA4 showed a 2-fold increase as compared to 0 hours with 23.4 U mg^−1^ protein. However, their activities decreased at 24 hours; the lowest value was observed in SA1 plants with 6.6 U mg^−1^ protein, which was about 4-times higher than that of WT plants (Figure 4c). A consistent increase in APX activity was detected in all leaves, especially in SA1, with the activity level showing a 3-fold increased at 24 h as compared to 0 h; protein levels increased from 0.06 μmol · min^−1^ · mg^−1^ protein to 0.33 μmol · min^−1^ · mg^−1^ protein. The WT only showed an increase from 0.05 μmol · min^−1^ · mg^−1^ protein to 0.17 μmol · min^−1^ · mg^−1^ protein. At 24 hours, all transgenic plant leaves showed a significant increase in activity compared to the WT plant leaves (Figure 4d).

### Enhanced tolerance to methyl viologen (MV)-mediated oxidative stress in transgenic leaves

**Figure 5:**
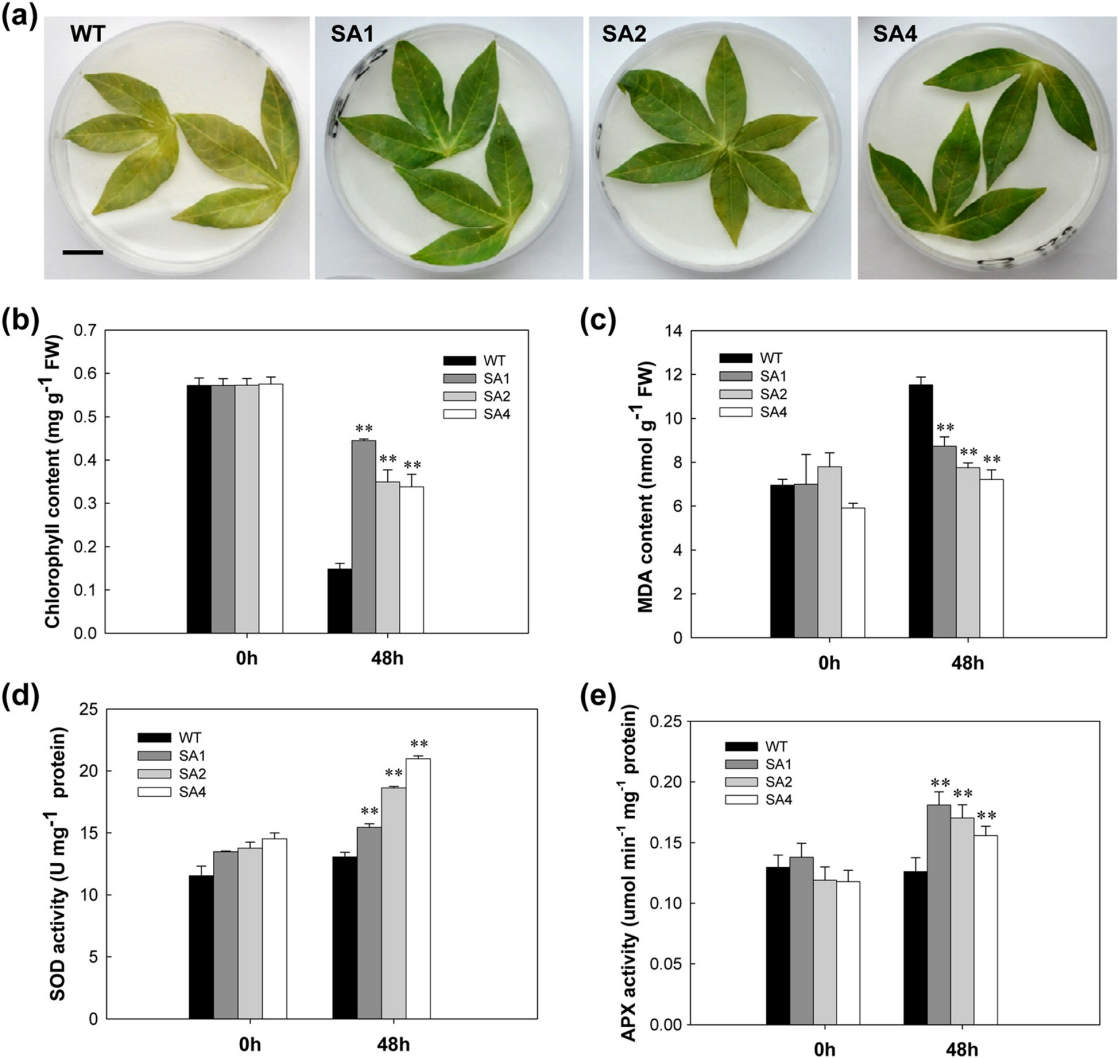
**Enhanced tolerance to methyl viologen (MV)-mediated oxidative stress in SA transgenic leaves. (a)** 100 μM MV-treated leaves showing senescence phenotype of WT and transgenic plants. Scale bar = 0.5 cm. **(b)** and **(c)** The chlorophyll and malondialdehyde (MDA) contents in the first leaf of MV-treated and untreated plants **(d)** and **(e)** Changes in SOD and APX activities between WT and transgenic cassava during MV treatment. WT, wild-type control; SA1, SA2 and SA4, independent transgenic plant lines. Data presented as mean ± SD from triplicate independent measurements. **Significant at 1% level from WT by *t-test*.

The effect of methyl viologen (MV), a superoxide-generating herbicide, on cassava was determined by subjecting the leaves to 100 μM MV for 2 days. Compared to WT plants, the extent of chlorophyll loss due to MV was significantly less in transgenic leaves (Figure 5a). As shown in Figure 5b, the chlorophyll content was not significantly different between WT and transgenic plants prior to MV treatment. After MV treatment, the chlorophyll content of WT plant leaves decreased by 75%, whereas in SA1, SA2 and SA4 leaves, the chlorophyll loss was only 21%, 37% and 38%, respectively. MV treatment causes membrane-lipid peroxidation, leading to an increase in the malondialdehyde (MDA) content. After MV treatment, the MDA content in WT plants increased to a maximum of 45%, whereas in transgenic lines the MDA content increased by approximately 14% (Figure 5c). The average amount of MDA in WT was 11.5 nmol/g Fresh Weight (FW), which had 2.8 nmol/g FW more than SA1 line. These data suggests that transgenic cassava has less lipid peroxidation because of timely ROS scavenging. The impact of ROS scavenging enzymes in transgenic cassava was further confirmed when both SOD and APX activities were significantly enhanced after treatment (Figure 5d and e). The SOD and APX activity was approximately 1.5-times higher in transgenic plants as compared to WT plants. The highest activity of SOD reached 21 U/mg protein in SA4 line; and the highest value of APX was found in SA1 line with 0.18 μmol · min^−1^ · mg^−1^ protein. These data confirmed that improved performance of transgenic cassava leaves against oxidative stress is due to elevated SOD and APX activities of the ROS scavenging system.

### Improved cold tolerance of transgenic plants

**Figure 6:**
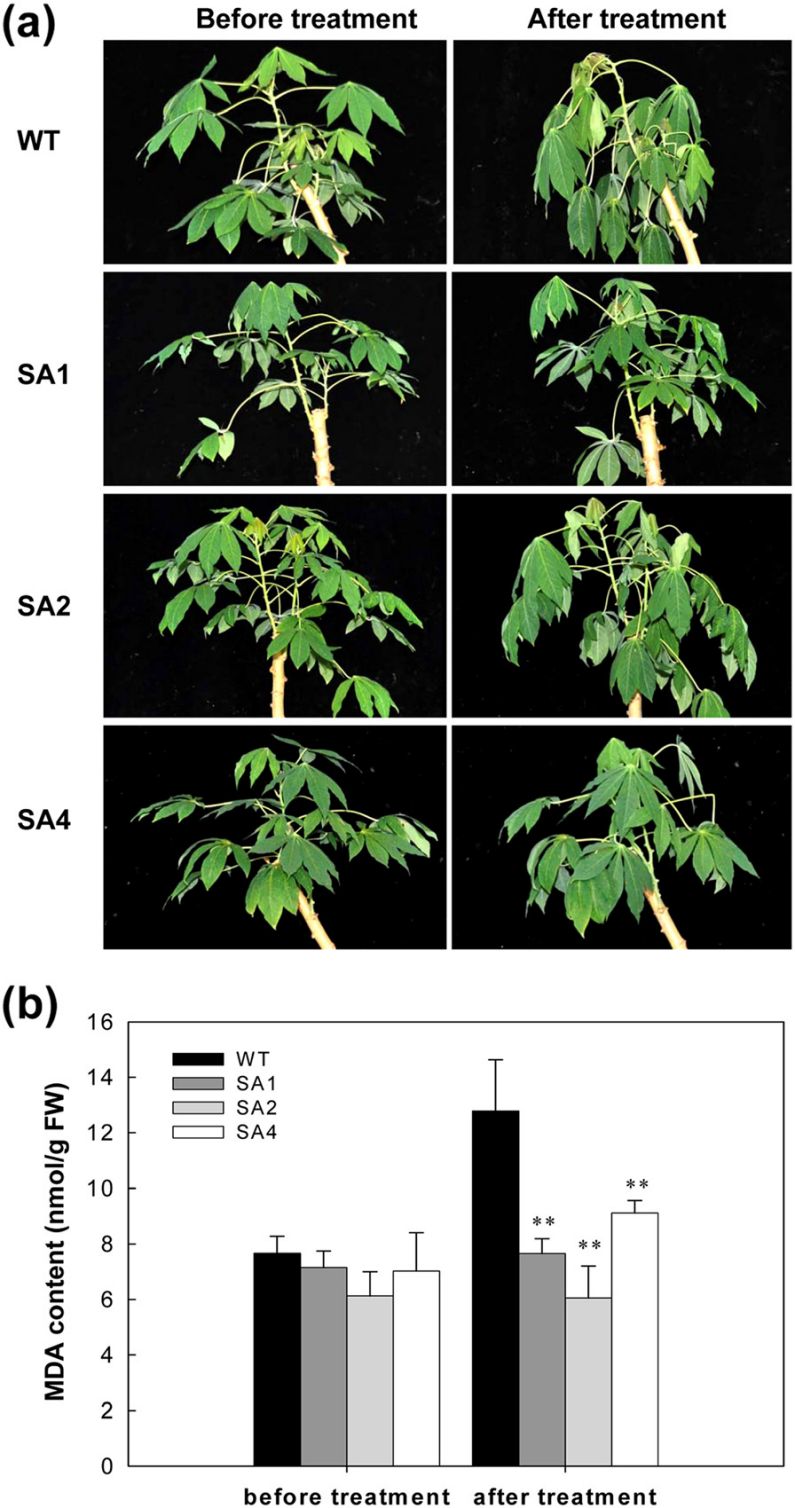
**Phenotypic changes of foliage (a) and the malondialdehyde (MDA) contents of the first fully expanded leaf (b) in low temperature stressed (4°C) two-month-old cassava after 2 h treatment.** WT, wild-type control; SA1, SA2 and SA4, independent transgenic plant lines.

Two-month-old plants were given a chilling treatment by transferring into a growth chamber at 4°C for 2 days. After the treatment, the WT plants wilted severely, whereas the transgenic lines were slightly affected, with fewer leaves wilting (Figure 6a). Among the three transgenic cassava lines, the SA1 line was the least affected by the chilling treatment. The level of MDA increased by 7%, 8%, and 20% in SA1, SA2, and SA4 lines after chilling stress, respectively. However, WT showed a 40% increase in MDA content, which was significantly higher than that of transgenic lines (Figure 6b).

**Figure 7:**
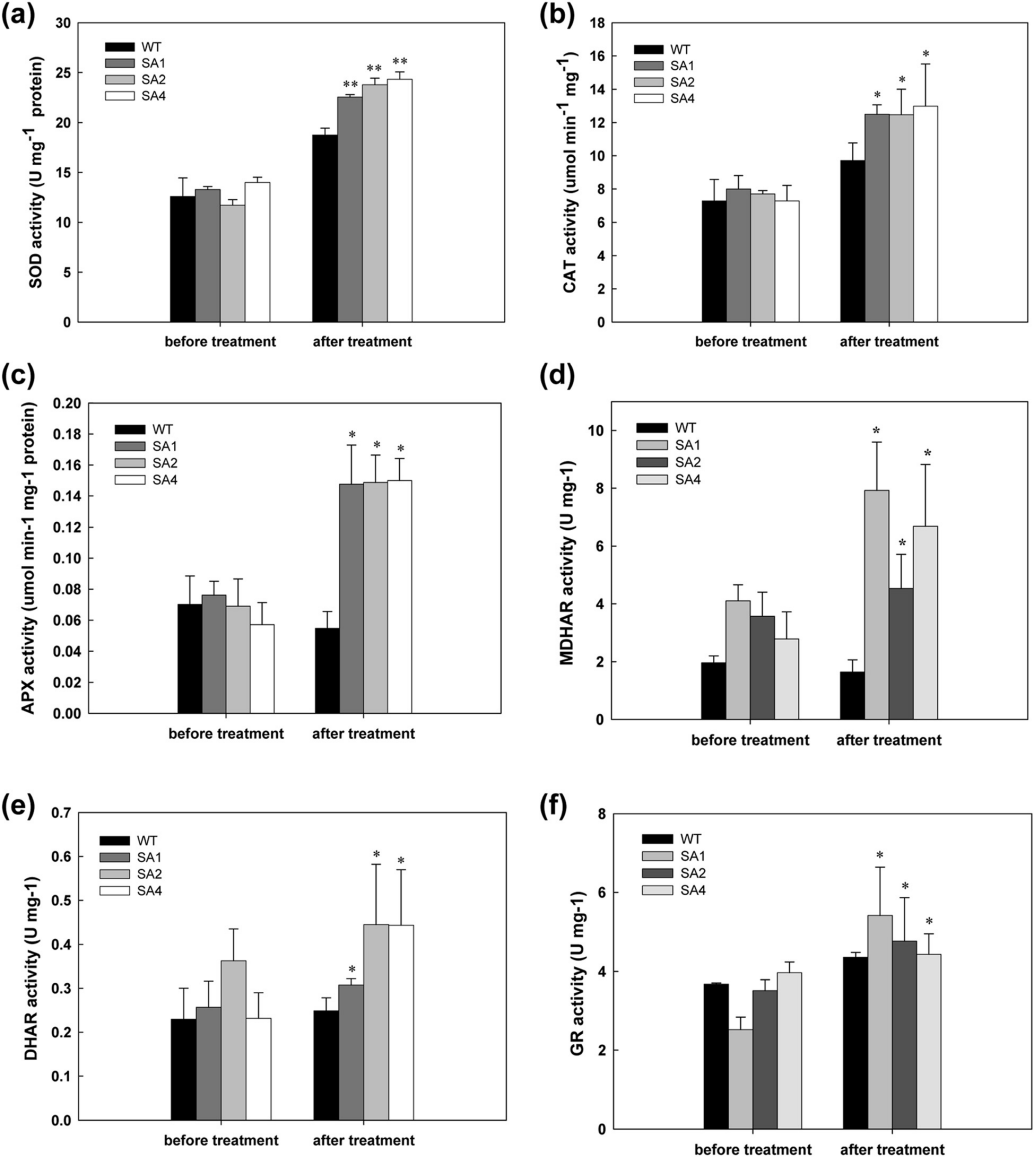
**Changes of enzymatic activities of SOD (a), CAT (b), APX (c), MDHAR (d), DHAR (e) and GR (f) between WT and transgenic cassava during cold treatment.** WT, wild-type control; SA1, SA2 and SA4, independent transgenic plant lines. Data presented as mean ± SD from triplicate independent measurements. *Significance at 5% level from WT by *t*-test, **Significance at 1% level from WT by *t*-test.

Other ROS-scavenging enzymes were monitored in both WT and SA transgenic cassava for their response to cold stress. Before treatment, both the WT and all the transgenic lines showed similar level of enzymatic activity. Upon cold treatment, increased activity of SOD, CAT, APX, MDHAR, DHAR and GR were confirmed in the transgenic lines as compared to the WT plants (Figure 7). The APX activity in SA lines showed >2-fold increase than WT (Figure 7c). The increase in SOD and CAT activities in transgenic lines was about 1.5-fold that of WT after treatment (Figure 7a, b). In the ascorbate cycle, MDHAR, DHAR and GR increased up to 43.6%, 30.6% and 28.6%, respectively (Figure 7d, e, f). However, no significant changes in enzyme activity were observed in WT before and after the treatment.

## Discussion

The cassava plant is generally considered to be cold-sensitive, and abiotic stresses, such as low temperature and salinity, dramatically affect their growth, thereby, leading to reduced productivity [41],[42]. Excessive ROS generation, which might result in hypersensitive response and cell death in cassava, has been identified as an important indicator of such conditions [5],[36]. Therefore, maintaining ROS homeostasis via ROS production and scavenging mechanisms is critical [36],[43]. Optimum regulation of ROS generation and scavenging by the mobilization of various pathways has been proposed as a vital mechanism for managing stress in cassava [5],[36], and our previous study had confirmed that increased resistance to abiotic stress could be achieved in transgenic cassava by over-expression of cytosolic Cu/ZnSOD and peroxisomal CAT1 [5],[7]. In this study, we showed that transgenic cassava with coupled expression of cytosolic *Me*Cu/ZnSOD and cytosolic *Me*APX2 leads to improved oxidative and chilling stress resistance through elevated ROS scavenging. Our studies confirmed that the response to abiotic stress can be improved in cassava by genetic engineering techniques that combine two different ROS-scavenging pathways, i.e., SOD/CAT and SOD/ASC-GSH cycle.

Most of ROS scavenging enzymes co-expressed targeted the chloroplast [26]–[28]. Recently, Faize et al. [29] and Diaz-Vivancos et al. [12] reported that over-expression of cytosolic SOD and APX in tobacco and plums improved drought and salt tolerance. An interesting question raised is the effectiveness of the stacked ROS-scavenging enzymes with various subcellular targeting strategies in plants, undoubtedly their individual important function in stress defense [43],[44]. Since the substrate for APX activity is H_2_O_2_ that is a by-product of SOD action, their individual functions should be combined to achieve a synergistic effect on stress tolerance. Indeed, upon chilling, the enzymatic activity of MDHAR, DHAR and GR in the ASC-GSH cycle in the transgenic cassava was significantly increased (Figure 7). We noticed that, comparing to the transgenic cassava overexpressed cytosolic SOD and peroxisomal CAT [6], the SA transgenic cassava showed less tolerance to post-harvest physiological deterioration of their storage roots (data not shown). We suppose that the PPD occurrence in cassava storage root is directly linked to the oxidization process in the subcellular organelles of parenchyma cells; the action of in the peroxisome enables the timely scavenging of excess ROS generated *in situ*.

Both H_2_O_2_ and MV treatment in the presence of light lead to the generation of superoxide radicals and H_2_O_2_ in chloroplasts and mitochondria of plants. *In vivo* imaging of ROS using the fluorescent probe rhodamine-123, a stain readily sequestered by active mitochondria and mesophyll protoplasts, or DAB staining of cassava leaves showed improved tolerance of transgenic cassava cells to oxidative stress caused by H_2_O_2_ and MV (Figures 3, 4). At the cellular level, the viability of the mesophyll protoplast and integrity of the mitochondrial were clearly indicated by their tolerance to H_2_O_2_ in transgenic cassava cells (Figure 3). These results indicate that transgenic plants expressing both cytosolic SOD and cytosolic APX are able to rapidly scavenge superoxide and hydrogen peroxide at the site of generation, as well as prevent the formation of hydroxyl radicals, the most toxic ROS, prior to their interaction with target molecules, as noted in our previous report [6]. The changes were observed not only in cytosolic antioxidant system but also in the chloroplasts and mitochondria, which implied that changes in the cytosolic antioxidant defense impacts the subcellular compartments, consistent with previous reports [7],[12],[29],[45]. Therefore, ROS scavenging system in plant cells is an integrative network for developing an antioxidant machinery through cytosol and subcellular organelle interactions.

Under cold conditions, non-transformed cassava plants showed signs of oxidative stress-induced cellular damage such as wilting, and increased lipid peroxidation of leaves (Figure 6). Cassava is very sensitive to low temperature; cold temperatures disrupt the metabolic balance of plant cells, resulting in enhanced production of ROS, e.g. H_2_O_2_[5]. The timely detoxification of ROS is necessary for the maintenance of the Calvin cycle and transpiration [18]. Transcriptome profiling of low temperature-exposed cassava showed an increase in transcripts and enzyme activities of ROS scavenging genes and the accumulation of total soluble sugars [5]. In the SA transgenic lines, we confirmed that the enzyme activity of SOD and APX were significantly higher than that of WT during cold treatment (Figure 7). Increased catalytic activity of APX eliminated the endogenous H_2_O_2_ via the ASC-GSH cycle, which involves GR, DHAR and MDHAR, to restore the cellular redox state, thus, suggesting that the performance of ascorbate-glutathione cycle is essential for the regulation of an efficient system for scavenging the accumulated ROS in leaves during chilling stress. This response was correlated with the up-regulation of APX activity and by maintenance of ASC-GSH redox pools in cold-acclimated plants [46].

A higher CAT activity was also observed in the SA lines under stress conditions. This increase seemed to be involved in the removal of excess H_2_O_2_. Dong et al. [47] reported that peroxisomal metabolism responded to cold regulation through ROS by increasing the H_2_O_2_ production in the peroxisome [47]. H_2_O_2_ could also diffuse through the peroxisomal membrane into the cytosol [48], thus increasing the risk of oxidative damage in this compartment. It suggests that ROS scavenging system in cassava is a complex network involving multiple components of ROS production, turnover and scavenging as well as their cross-interactions.

## Conclusions

In conclusion, our results show the important role of cytosolic *Me*Cu/ZnSOD and cytosolic *Me*APX2 in cassava in improving ROS scavenging, thereby leading to reduced H_2_O_2_ accumulation and improved abiotic stress resistance. The results also confirm that the transgenic approach is effective in improving the stress resistance in cassava via proper gene stacking of ROS scavenging enzymes.

## Methods

### Plasmid constructions, cassava transformation and phenotype evaluation of transgenic cassava

The cDNA amplification of cassava *Cu/ZnSOD* (GenBank accession no. AY642137) and cassava *APX2* (GenBank accession no. AY973622) was determined by PCR using primers covering the full length of transcripts. The PCR fragment was sequenced and cloned into the binary vector pCAMBIA1301 under the control of vascular-specific promoter p54/1.0 promoter (GenBank accession no. AY217353.1) [40] and the ubiquitous CaMV 35S promoter, respectively to generate pC-P54::MeCu/ZnSOD-35S::MeAPX2 (Figure 1a). The plasmid was mobilized into *Agrobacterium tumefaciens* strain LBA4404 for cassava transformation using friable embryogenic callus of cultivar TMS60444. Embryogenic callus induction of cassava TMS60444, *Agrobacterium*-mediated genetic transformation and plantlet regeneration were performed by the methods described by Zhang et al. [49].

The uniform stem cuttings of field-grown cassava plants were used for phenotype and yield evaluation. Ten stem cuttings per transgenic line and WT were planted in late spring of 2012 in Wushe Plantation for Transgenic Crops, Shanghai, China, and harvested in early November, 2012. The performance of field plants was recorded regularly till harvest.

### Subcellular localization

The *MeAPX2* genes tagged with green fluorescent protein (GFP) were cloned into pCAMBIA1301, and the resulting constructs were used to transform *Agrobacterium tumefaciens* LBA4404. To identify the subcellular localization of the gene, *N. benthamiana* leaves were agroinfiltrated with the *Agrobacterium* strains harboring pC-35::MeAPX2-eGFP binary vectors, respectively, by the protocol described previously [50]. At 36 hours post-infiltration, the transiently transformed leaves were observed under a confocal microscope (FluoView FV1000, Olympus, Japan).

### Southern blot and Real-time RT-PCR analyses of transgenic cassava plants

Southern blot analysis entailed digestion of the genomic DNA (20 μg) of cassava leaves of *in vitro* plants with *Xba*I, followed by 0.8% (w/v) agarose gel electrophoresis and subsequent transfer to a positively charged nylon membrane (Roche, Mannheim, Germany). The PCR fragment of *MeAPX2* (1 Kb) was labeled with digoxigenin (DIG) using the PCR DIG Probe Synthesis Kit (Roche, Mannheim, Germany). Hybridization and detection were performed by using the DIG-High Prime DNA Labeling and Detection Starter Kit II (Roche, Mannheim, Germany), according to the manufacturer’s instructions.

Gene expression was analyzed by real-time RT-PCR (qRT-PCR) of transgenic plants. Briefly, total RNA was extracted from fresh cassava leaves of *in vitro* plants using the RNA Plant plus Reagent (Tiangen, Beijing, China) essentially as described previously [51]. The RNA samples were digested with DNase I and the first strand of cDNA was synthesized from 5 μg total RNA from each sample using M-MLV reverse transcriptase (Toyobo, Osaka, Japan). qRT-PCR was carried out using the Bio-Rad CFX96 thermocycler SYBR Green I Master Mix (Toyobo, Osaka, Japan) according to the manufacturer’s protocol, under the following PCR conditions: 95°C for 1 minute, followed by 40 cycles at 95°C for 15 seconds, 60°C for 15 seconds and 72°C for 20 seconds. The qRT-PCR primers were designed using Primer 3 Plus Software (http://www.primer3plus.com). The primers were: *MeCu/ZnSOD* (forward 5′- ATGTTCATGCCCTTGGAGAC -3′ and reverse 5′- GATCACCAGCATGACGAATG -3′), *MeAPX2* (forward 5′- CATTGATAAGGCCAGGAGGA -3′ and reverse 5′- TTGTTAGCAGCATGACCCTG - 3′), and *β-actin* (forward 5′- TGATGAGTCTGGTCCATCCA -3′ and reverse 5′- CCTCCTACGACCCAATCTCA -3′). Fold changes of RNA transcripts were calculated by the 2^-ΔΔCt^ method [52] with *β-actin* as an internal control [6]. A detailed method for qRT-PCR analysis is available in Additional file 3.

### Isolation and viability assay of cassava mesophyll protoplasts

*In vitro*-cultured cassava seedlings were grown at 25°C with light at 200 μmol · m^−2^ · s^−2^ and a photoperiod 16 hour/8 hour (light/dark). Protoplasts were isolated from the mature leaves of 30-day-old sterile plants, as described by Anthony et al. [53]. Leaves were sliced into 2 mm to 3 mm thin strips and incubated in digestion mixture (pH 5.8) containing 9% (w/v) mannitol, 1% Cellulose R-10 (Yakult Honsha Co., Tokyo, Japan), 1.5% macerozyme R-10 (Yakult Honsha Co., Ltd., Tokyo, Japan) and 25 mM morpholine ethanesulfonic acid (MES) in cell protoplast washing (CPW) medium (27.2 mg of KH_2_PO_4_, 101 mg of KNO_3_, 1480 mg of CaCl_2_ · 2H_2_O, 246 mg of MgSO_4_ · 7H_2_O, 0.16 mg of KI, 0.025 mg of CuSO_4_ · 5H_2_O per liter). After purification, the protoplasts were resuspended in CPW solution supplemented with 9% mannitol (CPW9M, pH 7.0) to a final concentration of 2 × 10^5^ cells/mL.

Protoplast viability was determined by fluorescein diacetate (FDA) staining [54]. The purified mesophyll protoplasts were treated for 5 minutes with CPW 9 M solution (pH 7.0) supplemented with 1 M H_2_O_2_ for 5 min, and then stained with FDA at a final concentration of 0.01%. Stained protoplasts were observed for fluorescence under a fluorescent microscope (Nikon TE2000-S, Japan). The viable percentage = (the number of protoplasts with green fluorescence)/(the number of total mesophyll protoplasts) × 100%.

### Analysis of mitochondrial integrity

Mitochondrial integrity was measured by rhodamine 123 (Rh 123, Molecular Probes-Invitrogen CA, USA) fluorescence [55] after cassava mesophyll protoplasts were treated with 1 M H_2_O_2_ for 5 min. Fluorescence was detected using a confocal laser scanning microscope (FluoView FV1000, Olympus, Japan) with Ex/Em of 488 nm/515 nm. In each sample, 20–25 cells were scanned and viewed.

### Treatment with MV, H_2_O_2_ and cold

Fully expanded, healthy leaves were excised from the one-month-old plants in greenhouse and put in 10 cm-diameter petri dishes with different chemical solutions. For the MV treatment, leaves were allowed to float on 50 mL of 100 μM MV, and for the H_2_O_2_ treatment, leaves were exposed to 50 mL of 0.5 M H_2_O_2_. Leaves were incubated at 25°C under light conditions.

Four-week-old seedlings of transgenic and the control plants were transplanted into pots (30 cm in diameter, 45 cm in height) and grown in a greenhouse (16 h days, 30°C days and 22°C nights). Two-month-old plants were subjected to stress conditions. For cold treatment, plants with a uniform growth status were transferred to a chamber and incubated at 4°C for 48 hours under weak light (cool-white fluorescent light at approximately 35 μmol · m^−2^ · s^−1^).

### Determination of chlorophyll content and lipid peroxidation

Chlorophyll was isolated from WT and transgenic leaf segments according to the procedure described by Arnon et al. [56]. One-gram leaf disks from the one-month-old plants were homogenized in 10 mL of absolute ethyl alcohol and the homogenate was centrifuged at 3500 × *g* for 5 minutes. The supernatant was retained and the absorbance was recorded at 663 nm and 646 nm using a Nano-Drop spectrophotometer (Thermo Scientific, Scientific, Wilmington, DE, USA). Lipid peroxidation in leaf tissues was measured in terms of MDA content in the samples, according to method described by Heath and Packer [57]. One-gram leaves were homogenized in 10 mL of 10% (w/v) trichloroacetate (TCA) and centrifuged at 10,000 × *g* for 10 minutes. Thereafter, 2 mL of 10% trichloroacetic acid containing 0.67% (w/v) thiobarbituric acid was added to 2 mL of the supernatant. The mixture was boiled for 15 min, quickly cooled on ice, and centrifuged at 10,000 × *g* for 5 minutes. The absorbance of the supernatant was recorded at 532 nm and corrected for non-specific turbidity by subtracting the absorbance at 600 nm using a Nano-Drop spectrophotometer (Thermo Scientific).

### Determination of H_2_O_2_ content and 3,3′-diaminobenzidine staining

After determining the lipid peroxidation as above, the quantitative measurement of H_2_O_2_ in the leaves was carried out according to the method reported by Velikova et al. [58]. The 3,3′-diaminobenzidine (DAB) staining method was used to qualitatively detect the H_2_O_2_ generation in leaves after treatments, using the method described by Thordal-Christensen et al. [59].

### Enzyme assays

SOD isoenzyme was analyzed by separating the protein extracts in 10% native polyacrylamide gel with a 4% stacking gel in standard tris-glycine buffer (pH 8.3). Samples were electrophoresed at 100 V through the stacking gel for 20 min and 120 V through the separating gel for 60 min. After electrophoresis, the gel was immersed in 0.1% (w/v) nitroblue tetrazolium (NBT) solution for 15 min, briefly washed in ddH_2_O, then immersed in 100 mM potassium phosphate buffer (pH 7.0) containing 0.028 mM riboflavin and 28 mM TEMED (N,N,N#,N#-tetramethyl-ethylenediamine) for another 15 min. Gels were briefly washed in ddH_2_O and illuminated on a light box, with a light intensity of 30 mE · m^−2^ · s^−1^ for 15 min to initiate the photochemical reaction [60].

SOD activity was detected according to the method of Beauchamp and Fridovich [61]. A 3-mL reaction mixture contained 50 mM potassium phosphate buffer (pH 7.8), 13 mM methionine, 75 mM nitroblue tetrozulium (NBT), 2 mM riboflavin, 0.1 mM EDTA, and 100 mL enzyme extract. The reaction was initiated by placing the tubes under light intensity of 5000 lx. The absorbance was measured at 560 nm in a spectrophotometer and one unit of SOD is defined as the amount required to inhibit the photo reduction of NBT by 50%.

To analyze the APX isozymes, the protein extracts were separated in 10% native polyacrylamide gels with a 5% stacking gel in standard tris-glycine buffer with 2 mM ascorbate (pH 8.3). Samples were electrophoresed under conditions similar to the SOD isozyme electrophoresis. After electrophoresis, the gels were immersed in 50 mM potassium phosphate buffer, pH 7.0, containing 2 mM ascorbate for 10 min; then in 50 mM potassium phosphate buffer, pH 7.0, containing 4 mM ascorbate and 1 mM H_2_O_2_ for 20 min. After rinsing in water, the gels were stained in 50 mM potassium phosphate buffer, pH 7.8, containing 14 mM TEMED and 2.45 mM NBT for 10–30 min [62].

APX activity was determined as described by Nakano and Asada [63]. The reaction mixture contained 50 mmol/L potassium phosphate, pH 7.0, 1 mmol/L ascorbic acid (AsA), 2.5 mmol/L H_2_O_2_ and enzyme source (ca 15 μg protein) in a final volume of 2 mL at 25°C. Ascorbate oxidation was measured spectrophotometrically by a decrease of A290, using the absorption coefficient of 2.8 mM^−1^ · cm^−1^.

The activities of monodehydroascorbate reductase (MDHAR, EC 1.6.5.4), dehydroascorbate reductase (DHAR, EC 1.8.5.1), and GR were assayed as described previously [64]–[66]. Protein was estimated according to Bradford [67].

### Statistical analyses

All data were represented as mean ± SD from at least three independent experiments with three replicates. Statistical analysis was done using SPSS 15.0 for Windows (SPSS, Chicago, IL), with two-tailed Student’s *t*-tests for measuring significance. Double asterisks indicate significant differences between transgenic lines and WT at 1% level, one asterisk stands for at 5% level.

## Competing interests

The authors declare that they have no competing interests.

## Authors’ contributions

JX carried out the transgenic cassava production, molecular and physiological analysis, and wrote the manuscript. JY, XD and YJ partially participated in the experiment and provided helpful suggestions. PZ was responsible for the overall concept, experimental design, data analysis, and revising this manuscript. All authors read and approved the manuscript.

## Additional files

## Supplementary Material

Additional file 1: **Subcellular localization of MeAPX2::GFP fusion protein and GFP control in*N. benthamiana*epidermal cells.** Scale bar = 50 μm. Click here for file

Additional file 2: **Yield of fresh storage roots in field-grown (5 months) wild type (WT) and SA transgenic plant lines.** No significant difference was found by *t*-test (*p* < 0.05). Click here for file

Additional file 3: Supplemental methods for qRT-PCR analysis. Click here for file
