# Supplementary figures and images for: Increased expression of native cytosolic Cu/Zn superoxide dismutase and ascorbate peroxidase improves tolerance to oxidative and chilling stresses in cassava (Manihot esculenta Crantz)
Source: BMC Plant Biol. 2014 Aug 5;14:208. doi: 10.1186/s12870-014-0208-4 (PMC4236755; doi:10.1186/s12870-014-0208-4)

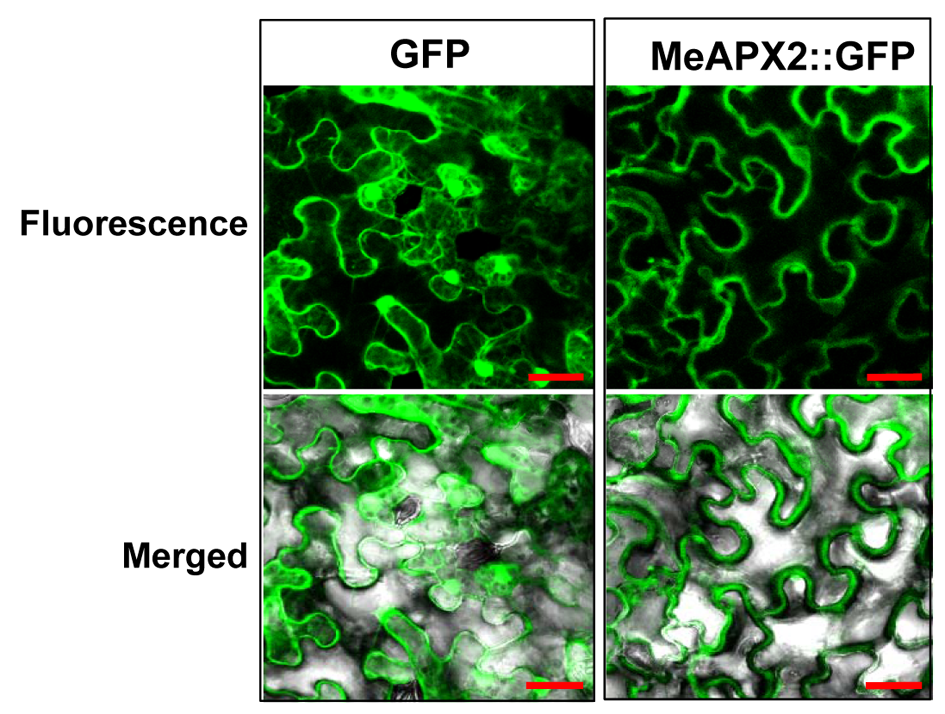

Supplement: Additional file 1: — Subcellular localization of MeAPX2::GFP fusion protein and GFP control inN. benthamianaepidermal cells. Scale bar = 50 μm. [file s12870-014-0208-4-S1.tiff]

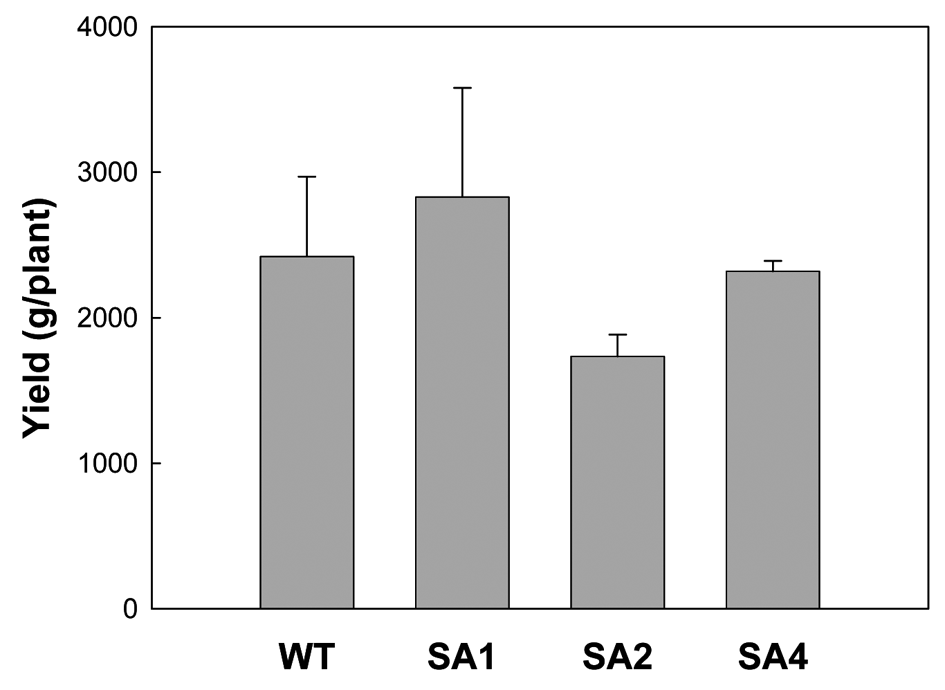

Supplement: Additional file 2: — Yield of fresh storage roots in field-grown (5 months) wild type (WT) and SA transgenic plant lines. No significant difference was found by t-test (p < 0.05). [file s12870-014-0208-4-S2.tiff]
