# Supplementary material for: Increased expression of native cytosolic Cu/Zn superoxide dismutase and ascorbate peroxidase improves tolerance to oxidative and chilling stresses in cassava (Manihot esculenta Crantz)
Source: BMC Plant Biol. 2014 Aug 5;14:208. doi: 10.1186/s12870-014-0208-4 (PMC4236755; doi:10.1186/s12870-014-0208-4)
Supplement: Additional file 3: — Supplemental methods for qRT-PCR analysis. [file s12870-014-0208-4-S3.docx]

**Additional file 3:**

**Supplemental methods for qRT-PCR analysis**

The fluorescence-based real-time quantitative reverse transcription polymerase chain reaction (qRT-PCR) was used to analyze the expression level of cassava endogenous genes as well as transgenes. The qRT-PCR performance was conducted according to the MIQE guidelines described by Bustin et al. (2009) [1]. A producible protocol of RNA extraction from cassava leaves and storage roots using the RNA Plant plus Reagent (Tiangen, Beijing, China) was used. Briefly, 0.1 g tissues of leaves or storage roots were grinded in a mortar using liquid nitrogen and quickly transfer the frozen powder to the prewarmed extraction buffer (0.6 ml) and mix completely by inverting the tube. After adding 0.5 mL of chloroform, mixing well and centrifuging at 12,000 rpm for 10 min at 4°C, the very viscous supernatant was transferred to a new tube, added 0.1 mL 5 M NaCl and 0.3 mL chloroform, mixed well and centrifuged at 12,000 rpm for 10 min at 4°C. The upper phase was transferred to a new RNAase-free tube and then added equal volume of isopropanol. After mixing well, the tube was store at room temperature for 10 min. The RNA was recovered by means of centrifugation at 12,000 rpm for 10 min at 4°C. After completely discarding the viscous supernatant, the pellet was washed with 75% ethanol to remove the remaining mucilage, and air- dried for 2-3 min. The total RNA was dissolved in 30-50 µL of DEPC-treated water and stored at -70°C until use. RNA quantification was carried out by the use of NanoDrop and running 1% agarose gels.

In the reverse transcription, the first strand of cDNA was synthesized from 5 µg total RNA from each sample using M-MLV reverse transcriptase (Toyobo, Osaka, Japan). RT-PCR conditions were: 95°C for 10 minute, followed by 30 cycles at 95°C for 30 seconds, 60°C for 30 seconds and 72°C for 30 seconds. RT-PCR of cassava actin was done to detect the efficiency of reverse transcription.

For qRT-PCR primers, the primers were designed by the use of software “Primer 3 Plus” (http://www.primer3plus.com). Two or three pairs of primers were designed for each gene in the pre-experiment and chosen the best primer pair which had no primer dimers in PCR reaction for qRT-PCR.

qRT-PCR reactions were set up in a final volume of 20 µL reaction containing 10 µL of 2×SYBR Master Mix (QPK-201, TOYOBO, Osaka, Japan), 50 ng of cDNA, 400 nmol/L of forward primer and reverse primer. qRT-PCR was carried out using the Bio-Rad CFX96 thermocycler SYBR Green I Master Mix (Toyobo, Osaka, Japan) according to the manufacturer’s protocol, under the following PCR conditions: 95°C for 1 minute, followed by 40 cycles at 95°C for 15 seconds, 60°C for 15 seconds and 72°C for 20 seconds. Each sample had three repetition and fold changes of RNA transcripts were calculated by the 2^-ΔΔCt^ method [2] with β-actin as an internal control as described in M&M.

**References**

1. Bustin SA, Benes V, Garson JA, Hellemans J, Huggett J, Kubista M, Mueller R, Nolan T, Pfaffl MW, Shipley GL, Vandesompele J, Wittwer CT: **The MIQE guidelines: minimum information for publication of quantitative real-time PCR experiments.** *Clinical chemistry*, 2009, **55**: 611-622.
2. Livak KJ, Schmittgen TD: **Analysis of relative gene expression data using real-time quantitative PCR and the 2^-ΔΔCt^ method.** *Methods* 2001, **25(4):** 402-408.
